# Supplementary material for: Altered microRNA Transcriptome in Cultured Human Liver Cells upon Infection with Ebola Virus
Source: Int J Mol Sci. 2021 Apr 6;22(7):3792. doi: 10.3390/ijms22073792 (PMC8038836; doi:10.3390/ijms22073792)
Supplement: Supplementary file 1 [file ijms-22-03792-s001.zip › Supplementary_File/C_ GO_Analysis_Results/16-30nt_go_Makona-96h-Huh7_vs_Control-96h-Huh7_up.mature_mirna_targets/MF_EnrichmentScore.pdf]

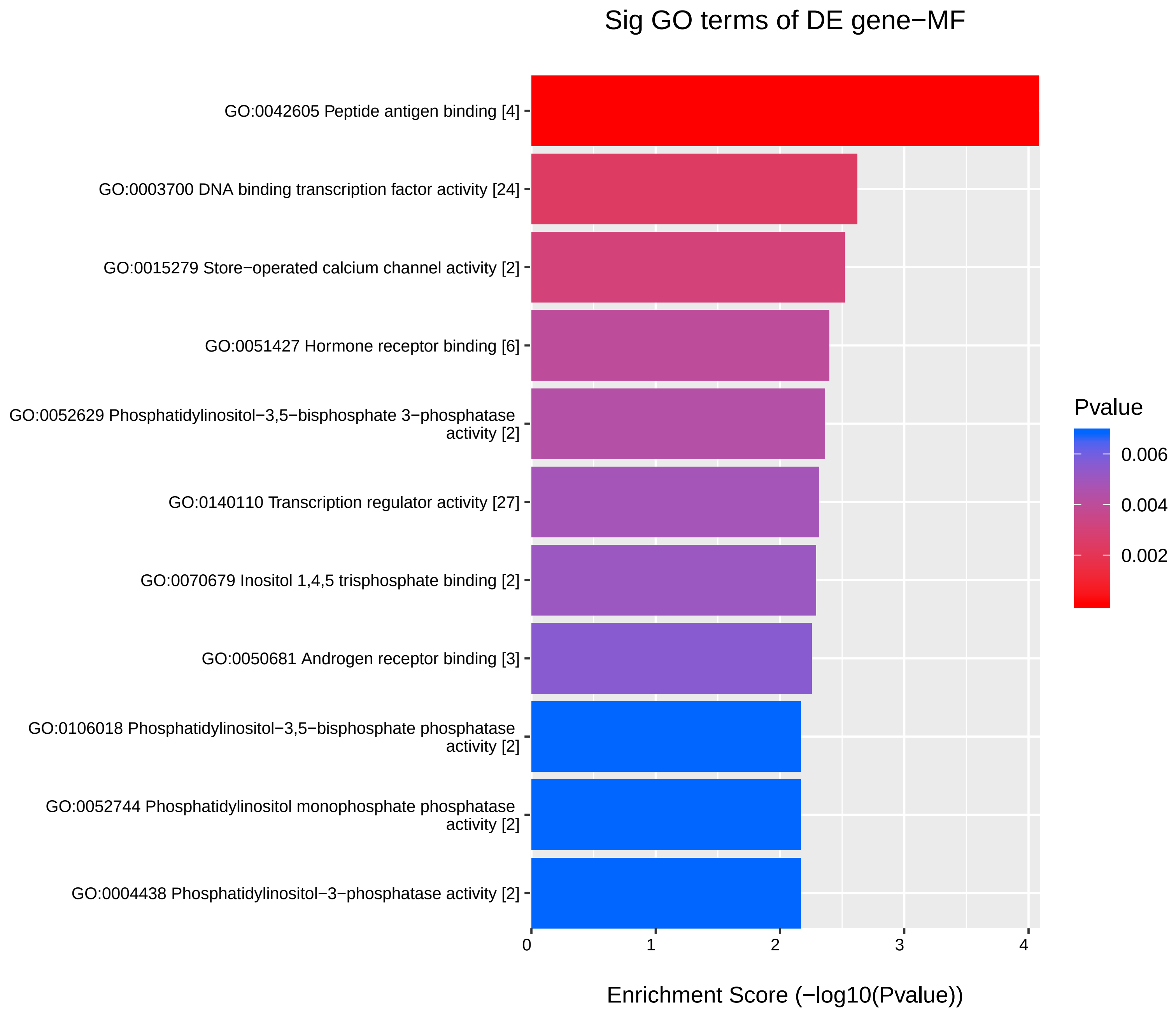

Sig GO terms of DE gene−MF
GO:0042605 Peptide antigen binding [4]
GO:0003700 DNA binding transcription factor activity [24]
GO:0015279 Store−operated calcium channel activity [2]
GO:0051427 Hormone receptor binding [6]
GO:0052629 Phosphatidylinositol−3,5−bisphosphate 3−phosphatase activity [2]
GO:0140110 Transcription regulator activity [27]
GO:0070679 Inositol 1,4,5 trisphosphate binding [2]
GO:0050681 Androgen receptor binding [3]
GO:0106018 Phosphatidylinositol−3,5−bisphosphate phosphatase activity [2]
GO:0052744 Phosphatidylinositol monophosphate phosphatase activity [2]
GO:0004438 Phosphatidylinositol−3−phosphatase activity [2]
0
1
2
3
4
Enrichment Score (−log10(Pvalue))
Pvalue
0.006
0.004
0.002
